# Salvage Surgery for a Recalcitrant Clavicular Nonunion Using the Masquelet Technique: A Case Report

**DOI:** 10.7759/cureus.78990

**Published:** 2025-02-14

**Authors:** Yo Kinami, Masahiro Horita, Koki Ogasa, Kazuo Fujiwara

**Affiliations:** 1 Department of Orthopaedic Surgery, Okayama City Hospital, Okayama, JPN; 2 Department of Orthopaedic Surgery, Okayama University Graduate School of Medicine, Dentistry and Pharmaceutical Sciences, Okayama, JPN

**Keywords:** alignment, anterior plate fixation, bone graft, clavicle fracture, clavicle nonunion, length, locking plate, masquelet method, recalcitrant nonunion, template

## Abstract

A 47-year-old man sustained an injury in a motorcycle accident and was transported to our hospital by ambulance. Radiography and computed tomography revealed a midshaft fracture of the left clavicle with multiple fragments and displacement. One week after the injury, anterior plate fixation was performed at our hospital using a locking plate with suture stabilization of the bone fragments. However, the initial plate fixation surgery failed, resulting in nonunion and necessitating plate removal. One year and 10 months post-injury, reconstructive surgery for the nonunion was attempted using the double-plate fixation method, with bone grafting. However, the plates were removed due to breakage and bone graft resorption.

Three years and six months post-injury, the patient requested surgery due to persistent dull shoulder pain, shoulder droop, and difficulty performing tasks requiring shoulder elevation, caused by pain from fragment irritation. Salvage surgery was performed using the Masquelet technique. During the first-stage surgery, a 3 cm bone defect was filled with a cement spacer after refreshing and drilling the fragment ends. Clavicle length and alignment were reconstructed using locking plate fixation, guided by a two-dimensional template based on an actual-sized clavicle image. Six weeks later, in the second-stage surgery, cancellous bone chips and β-tricalcium phosphate chips were grafted into the induced membrane. Four years and six months post-injury, bone union was achieved, and the patient attained full functional recovery and remained pain-free. This case highlights the potential of the Masquelet technique as a treatment option for recalcitrant clavicle nonunion.

## Introduction

Historically, midshaft clavicular fractures were treated conservatively due to their lower nonunion rates compared to surgical treatment [1]. However, recent multicenter randomized controlled trials (RCTs) have shown that operative treatment achieves a higher union rate and better functional outcomes than conservative treatment, particularly in cases of midshaft clavicle fractures with multifragmentary patterns or significant displacement [2-4]. A meta-analysis reported that the nonunion rate for operative treatment of midshaft clavicle fractures ranges from 0% to 3%, compared to 3%-20% for conservative treatment [5]. Despite these advantages, recent studies have identified construct failure rates of 1.2%-13.7%, with complications such as plate breakage, plate bending, and screw loosening [6-10].

The healing rate of reconstruction surgery after conservative treatment of clavicle nonunions is reported to be 87%-96% [11-13], whereas the healing rate of reconstruction surgery for nonunion following operative treatment remains unclear. Clavicle nonunions, which require more than one surgical procedure to achieve union, are termed recalcitrant [13]. Salvage surgery for recalcitrant clavicle nonunion presents significant challenges and may involve microsurgical techniques such as vascularized bone grafting [14,15].

The Masquelet technique is a two-stage surgical method used to reconstruct large defects in long bones. This approach accelerates bone graft maturation by encasing it in an induced membrane [16-18]. Although the technique is commonly used for femoral and tibial bone defects, there are few reports of its usefulness for clavicle bone defects.

Here, we report a case of salvage surgery using the Masquelet technique to treat recalcitrant clavicle nonunion that occurred following failed reconstruction surgery with double-plate fixation and bone grafting, after the initial operative treatment. The patient provided informed consent for the surgical procedure and the publication of this report.

## Case presentation

**Figure 1:**
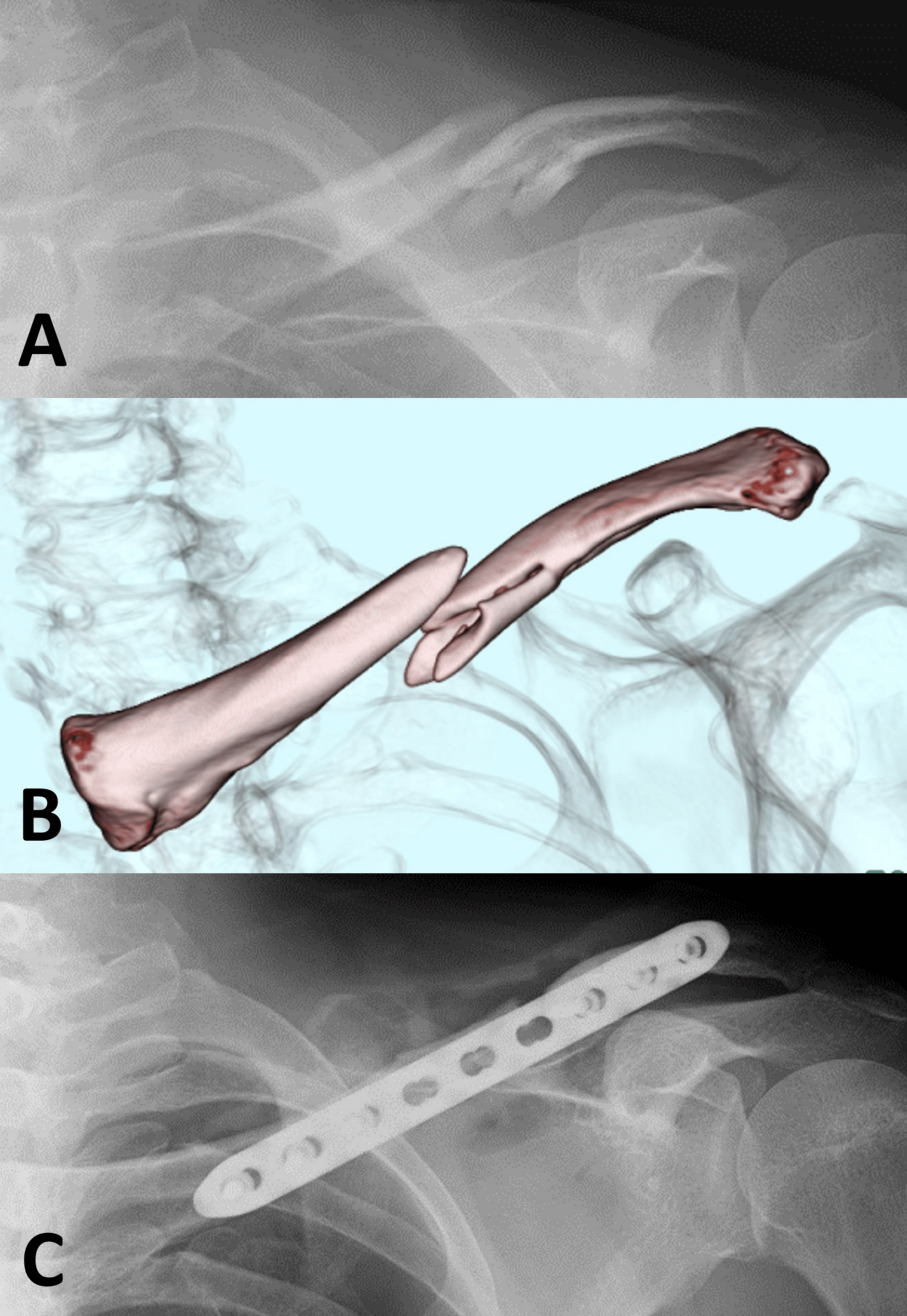
Injury and initial surgery X-ray imaging at the time of injury revealed a displaced midshaft fracture of the left clavicle (A), while three-dimensional computed tomography showed two fragments at the fracture site (B). Postoperative X-ray imaging showed anterior plate fixation using only cortical screws (C).

A 47-year-old man sustained an injury in a motorcycle accident and was transported to our hospital by ambulance. Radiography and computed tomography (CT) revealed a midshaft fracture of the left clavicle with multiple fragments and displacement (Orthopaedic Trauma Association (OTA) classification: 15-2C). The patient worked as a machine operator in a steel factory and had a dominant right hand. He was healthy, a non-smoker, non-obese, non-diabetic, and did not have a vitamin D deficiency. One week after the injury, open reduction and anterior plate fixation were performed using a locking plate (LCP small plate; Depuy-Synthes, Warsaw, IN, USA) with only cortical screws, along with circumferential suture stabilization of the bone fragments, at our hospital (Figure 1). Postoperatively, shoulder elevation was allowed without limitation based on pain tolerance, but only desk work and light duties were permitted at his workplace. Three months later, shoulder elevation was pain-free and full, allowing the patient to return to work fully. However, low-intensity pulsed ultrasound therapy was initiated to address the delayed union.

**Figure 2:**
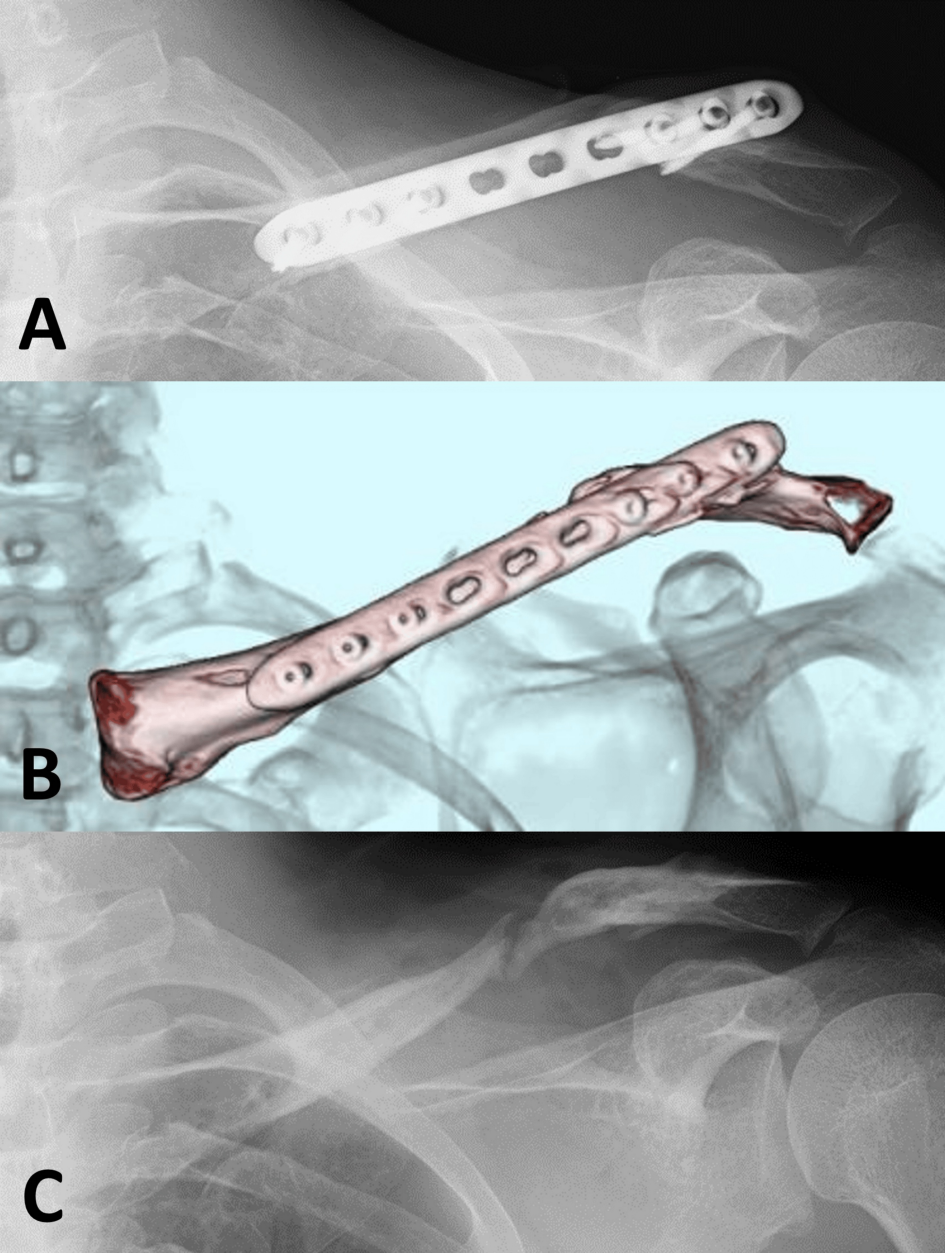
Nonunion after initial surgery X-ray imaging revealed protrusion of the distal end of the plate toward the skin (A), and three-dimensional computed tomography suggested a possible loosening of the distal screws (B) at one and a half years post-injury. An X-ray taken after plate removal revealed atrophic nonunion (C).

**Figure 3:**
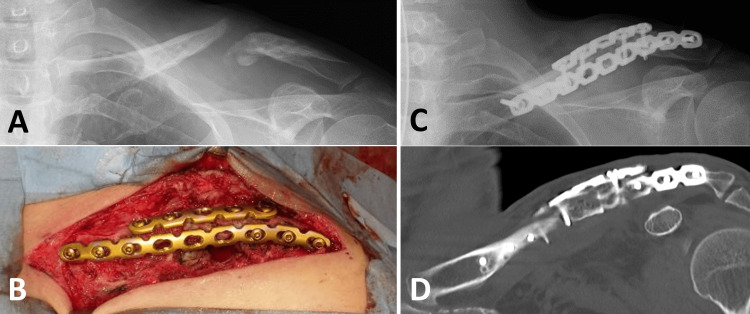
Reconstruction surgery for nonunion X-ray imaging showed protrusion of the proximal fragment toward the skin (A). Both the photograph and X-ray demonstrated double plate fixation and bone grafting using a tricortical block and cancellous chips (B, C). Computed tomography indicated adequate compression between the tricortical block and the fracture fragments (D).

One and a half years post-injury, the plate was removed due to pain caused by irritation at the plate ends, revealing nonunion (Figure 2). Despite this, the patient retained a full range of motion (ROM) in shoulder elevation. One year and 10 months post-injury, reconstructive surgery for nonunion was performed with double plate fixation (LCP small reconstruction plate; DePuy-Synthes) and bone grafting from the left iliac crest (Figure 3), as the patient experienced decreased shoulder ROM and pain from irritation at the proximal fragment end. Postoperatively, shoulder elevation was allowed without limitation based on pain tolerance, but only desk work and light duties were permitted at his workplace. Four months later, shoulder elevation was pain-free and full, allowing the patient to return to work fully.

**Figure 4:**
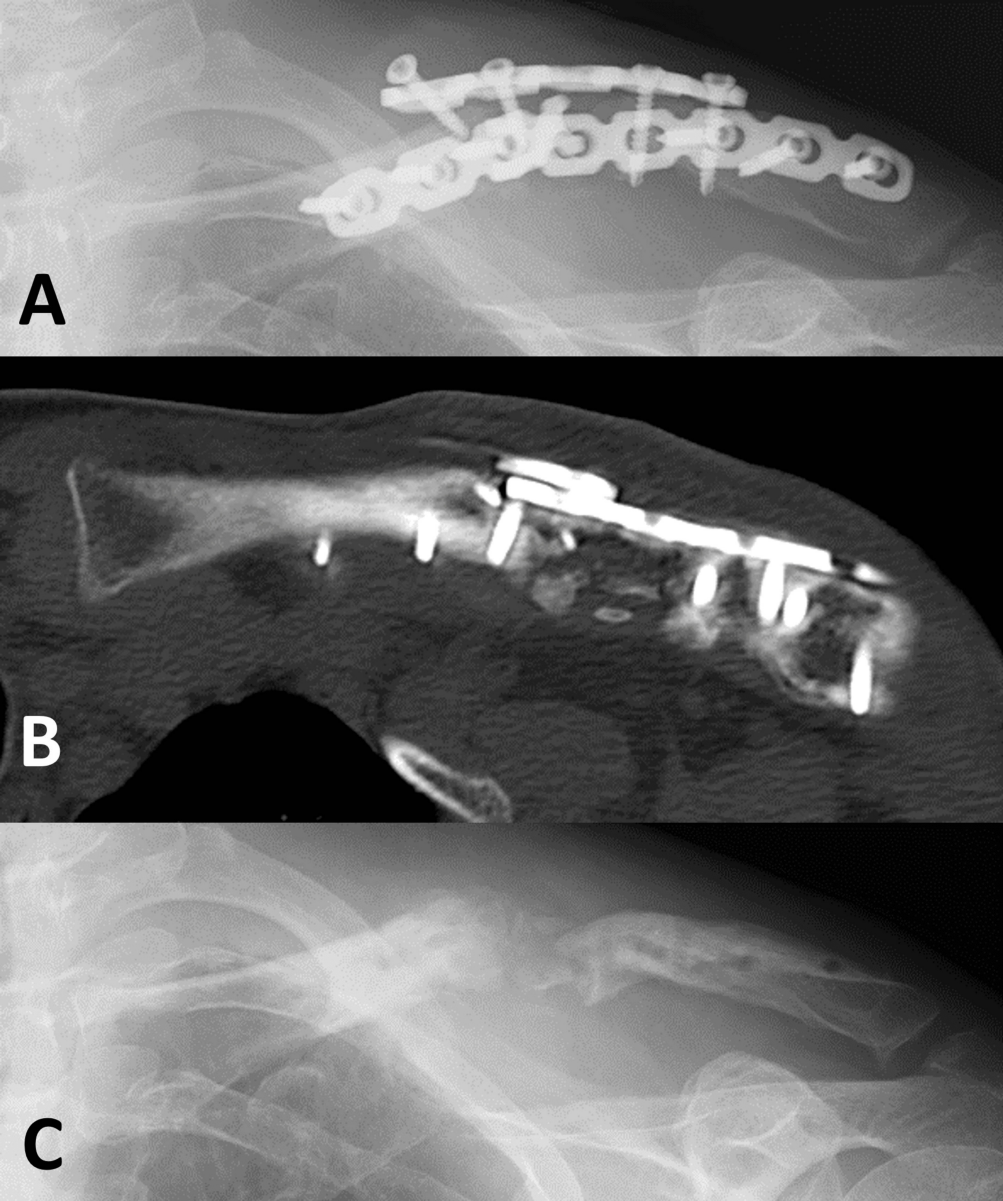
Plate breakage and bone graft resorption Plate breakage and bone graft resorption occurred six months after the reconstruction surgery (A, B). The plates were removed again (C), with no bacterial growth observed in the culture.

The plates were removed at two years and four months post-injury because of plate breakage and bone graft resorption, without bacterial infection (Figure 4). By two years and six months post-injury, the patient was pain-free, had recovered his full ROM in the shoulder, and returned to work, leading to the discontinuation of follow-up care.

**Figure 5:**
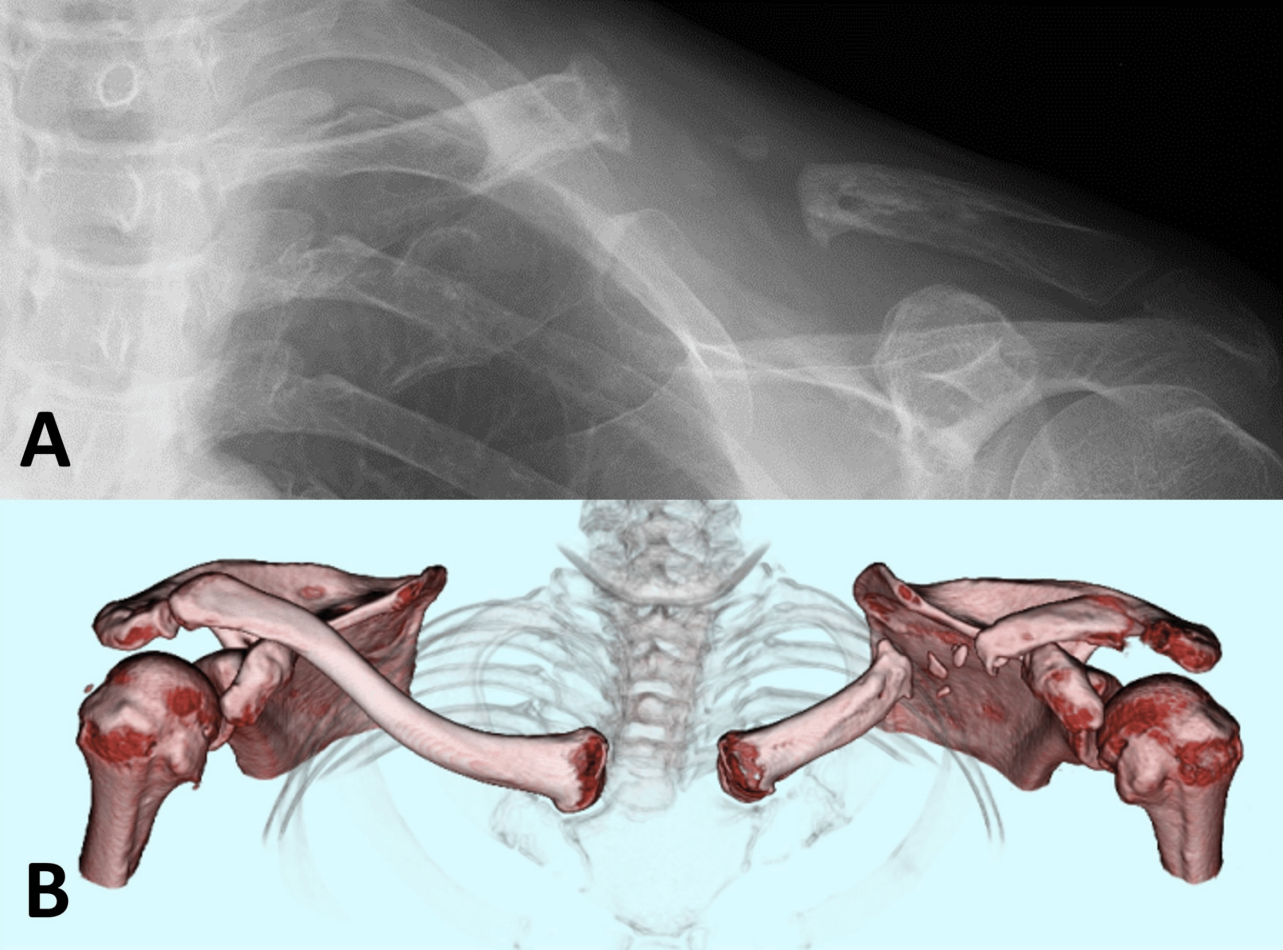
Recalcitrant nonunion X-ray imaging and three-dimensional computed tomography showed complete resorption of the bone graft and increased dissociation of the fracture fragments at three years and six months post-injury (A, B).

However, at three years and six months post-injury, the patient returned to the hospital with persistent dull shoulder pain, shoulder droop, and difficulty working in positions requiring shoulder elevation due to pain from fragment irritation. Active shoulder elevation was reduced to 135 degrees, and the DASH (Disabilities of the Arm, Shoulder, and Hand) score was 15.8 points. His employer warned that continued functional impairment would result in dismissal. Radiography and CT revealed complete bone graft resorption and increased fragment dissociation (Figure 5).

**Figure 6:**
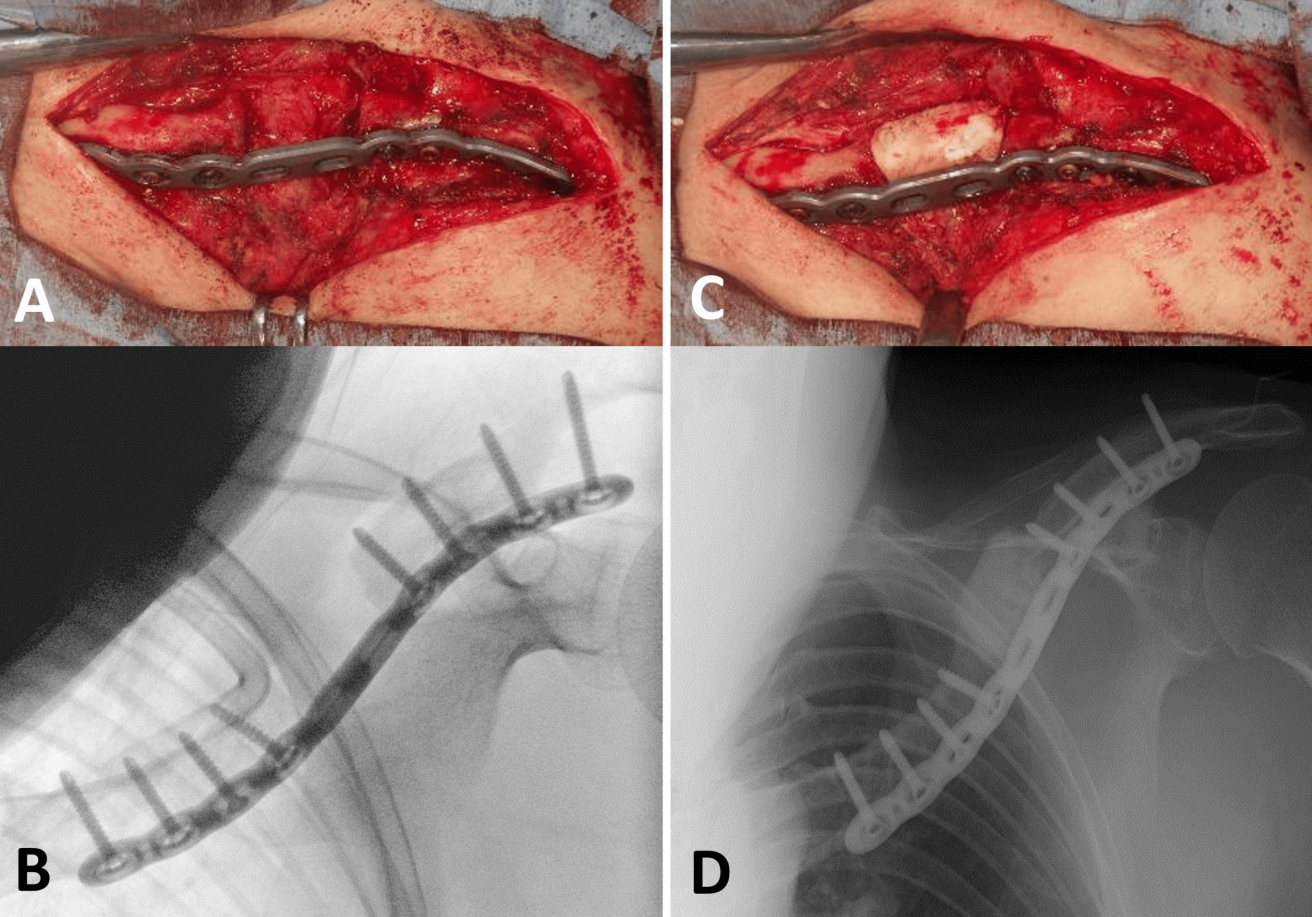
First stage of the Masquelet technique Anterior plate fixation was performed using an anatomical locking plate after refreshing and drilling the fragment ends (A, B). The bone defect was filled with a cement spacer containing antibiotics preventively, with a mixing ratio of 80 mg gentamicin per 40 g of cement (C, D). Clavicle length and alignment were restored using a plate bent according to the template.

**Figure 7:**
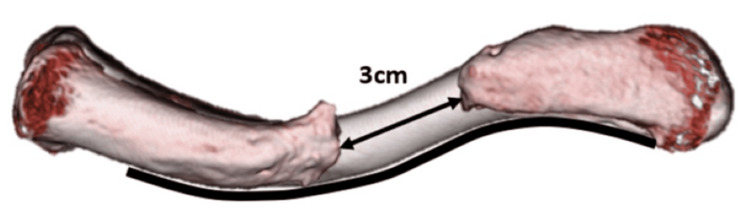
Two-dimensional template of a mirrored image of actual-sized normal clavicle and nonunion fragments The template was created using presentation software (Microsoft PowerPoint 2019). It was used to assess the size of the bone defect before surgery and to shape the plate to the anatomical contour during surgery. In this case, the bone defect measured 3 cm, and the plate was bent along the black curved line.

To address the nonunion, salvage surgery was performed using the Masquelet technique. In the first stage, anterior plate fixation was achieved using an anatomical locking plate (Variax clavicle anterior midshaft plate; Stryker, Kalamazoo, MI, USA) after refreshing and drilling the fragment ends. A 3 cm bone defect was filled with a cement spacer (Figure 6). Clavicle length and alignment were reconstructed using a two-dimensional (2D) template of a mirrored image of an actual-sized normal clavicle (Figure 7), created using presentation software (Microsoft PowerPoint 2019; Microsoft® Corp., Redmond, WA, USA).

**Figure 8:**
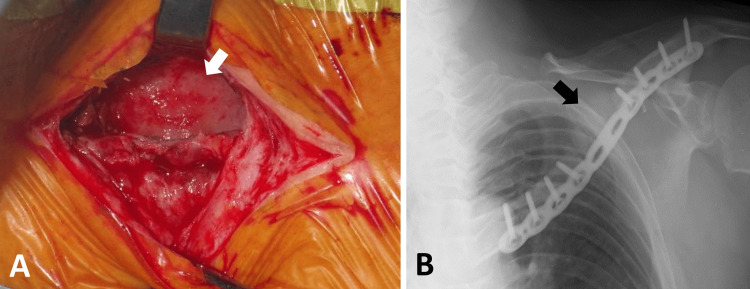
Second stage of the Masquelet technique Six weeks after the first stage procedure, the induced membrane (white arrow) was observed following the removal of the cement spacer (A). Cancellous bone chips and β-tricalcium phosphate chips (black arrow) were grafted into the induced membrane (B).

Six weeks later, cancellous bone chips from the left iliac crest and β-tricalcium phosphate (β-TCP) chips were grafted into the induced membrane (Figure 8). The left iliac crest, which had already been used as the donor site for reconstructive surgery, was selected again for grafting. This choice was made because the required graft volume was small, and using the right iliac crest as the donor site could lead to future problems, such as reducing the donor site available for bone grafting. However, the volume of cancellous bone chips alone was insufficient to fill the space within the induced membrane, as the surrounding cancellous bone had transformed into cortical bone following the previous surgery. To address this, β-TCP chips were mixed with cancellous bone chips, comprising one-third of the total volume.

**Figure 9:**
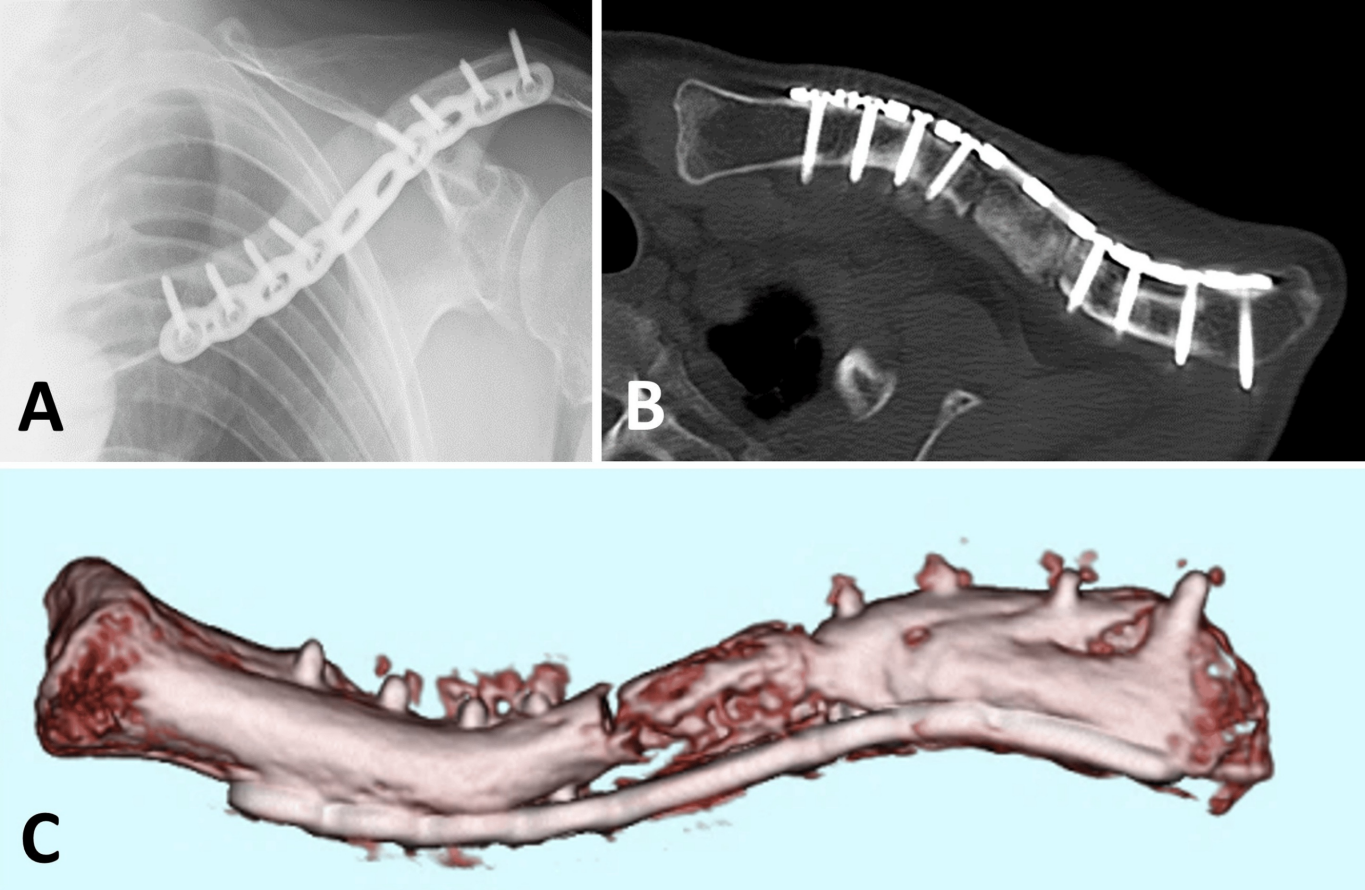
Three months after the second stage procedure The images show maintained alignment and maturation of the grafted bone (A, B, C).

**Figure 10:**
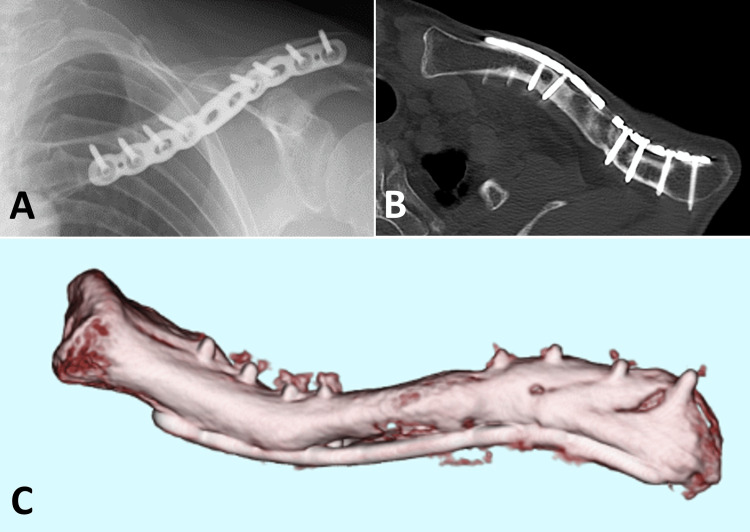
Nine months after the second stage procedure The images show that the grafted bone and both fragments were fully connected, achieving bone union (A, B, C).

Postoperatively, shoulder elevation was allowed without limitation based on pain tolerance, but only desk work and light duties were permitted at his workplace. Three months after the second-stage procedure, radiographs of the grafted bone showed signs of maturation (Figure 9). Six months later, shoulder elevation was pain-free and full, allowing the patient to return to work fully. Nine months after the second-stage procedure, the grafted bone and both fragments were fully connected, and bone union was achieved (Figure 10). Four years and six months post-injury, the patient had regained full shoulder ROM, was pain-free, and achieved a DASH score of 1.7 points, allowing him to return to his job at the same level as before the injury.

## Discussion

To the best of our knowledge, this case report is the first to document salvage surgery using the Masquelet technique for aseptic, recalcitrant nonunion of a clavicle shaft fracture more than three years post-injury.

The failure of the initial plate fixation surgery was probably caused by poor vascularity and unstable fixation. The suture stabilization of the bone fragments might have compromised the vascularity of the third fragment. Poor alignment and low stability were probable due to inadequate plate bending and the use of a bridging plate technique with only cortical screws (Figure 1). Consequently, the outcome of nonunion was an expected result (Figure 2). Atraumatic handling of the third fragment, adequate plate bending, and a bridging plate technique with locking screws were necessary for this fracture.

In reconstructive surgery for clavicular nonunion after conservative treatment, plate fixation alone is typically performed for hypertrophic nonunion, whereas plate fixation with bone grafting is used for atrophic nonunion. There is no significant difference in healing rates between locking plates and conventional plates, or between autogenous bone grafts and bone morphogenetic proteins (BMPs) [13]. The healing rate of reconstruction surgery for nonunion following operative treatment remains unclear, and a consensus on the treatment strategy has not yet been established. Therefore, the same reconstruction surgery strategy used for nonunion after conservative treatment was applied to this patient. Reconstruction surgery was performed using double conventional plate fixation with a tricortical bone block and cancellous bone chips harvested from the left iliac crest. The bone block was compressed three times using dynamic holes (Figure 3). Unfortunately, the grafted bone was resorbed, and the plate broke (Figure 4).

Clavicle nonunions requiring more than one surgical procedure to achieve union are termed recalcitrant [13]. Factors contributing to nonunion include poor vascularity, infection, diabetes, obesity, smoking, and insufficient vitamin D levels [19]. This patient did not exhibit any evidence of infection or smoking, nor was he diabetic, obese, or vitamin D deficient; therefore, poor vascularity may be the main factor contributing to the nonunion. The strategy for reconstruction surgery in atrophic nonunion following operative treatment of clavicle fractures may need to differ from that used for atrophic nonunion after conservative treatment, similar to the approach for recalcitrant nonunion.

Salvage surgery for recalcitrant clavicular nonunion is particularly challenging and may occasionally require vascularized bone grafts because of inadequate vascularity [14,15]. However, vascularized bone graft surgery is highly invasive and requires advanced microsurgical expertise. The Masquelet technique [16,17] is a two-stage reconstruction method for addressing large defects in long bones and can be performed using standard orthopedic surgical techniques. In the first stage, a cement spacer was used to fill the defect space, and in the second stage, cancellous bone was grafted into the space after removing the cement spacer, which was then wrapped in an induced membrane. An interval of six to eight weeks has been recommended between the two stages. The induced membrane secretes growth factors and BMPs that accelerate grafted bone maturation. The healing rate of bone defects treated using the Masquelet technique is approximately 86%, with numerous clinical and fundamental studies conducted by various institutions using this approach [18].

This technique is frequently used to heal bone defects in the femur and tibia but has rarely been applied to the clavicle [18]. While no reports exist on the use of the Masquelet technique for aseptic recalcitrant nonunions of clavicle shaft fractures, one study reported good results when applied to septic nonunion of the clavicle [20]. In our aseptic case, three months after the second step of this method, the grafted bone matured and consolidated without graft resorption (Figure 9B). The Masquelet technique can be useful for salvage surgery for aseptic recalcitrant clavicular nonunion.

The graft in the Masquelet technique matures through endochondral ossification, similar to conventional cancellous bone grafting, and is typically applied to long bones that grow and form via endochondral ossification. Therefore, the Masquelet technique has rarely been applied to flat bones, such as the clavicle, which grow and form through intramembranous ossification. However, in our patient, bone union using the Masquelet technique was successfully achieved in the clavicle. We hypothesize that the graft matures through endochondral ossification, while its fusion with the clavicular fragment occurs through intramembranous ossification. This, however, remains a topic for future research.

**Figure 11:**
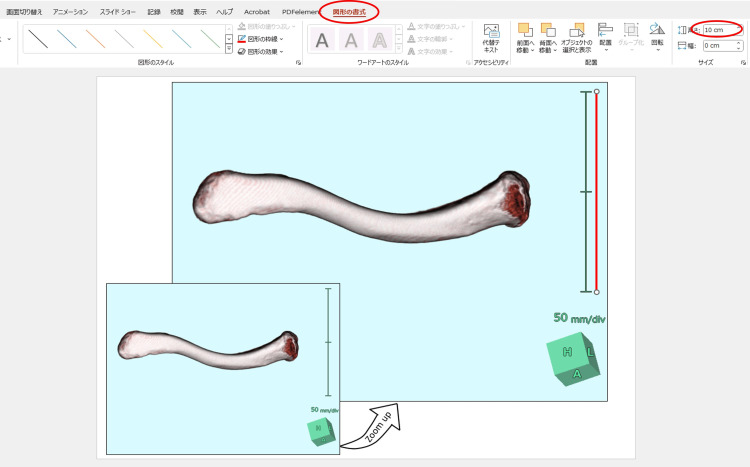
Actual sizing of three-dimensional computed tomography image The sizes of the normal clavicle image were adjusted to their actual size using the displayed scale and tools in their presentation software. The red line represents a length of 10 cm, while the image scale indicates a length of 100 mm.

**Figure 12:**
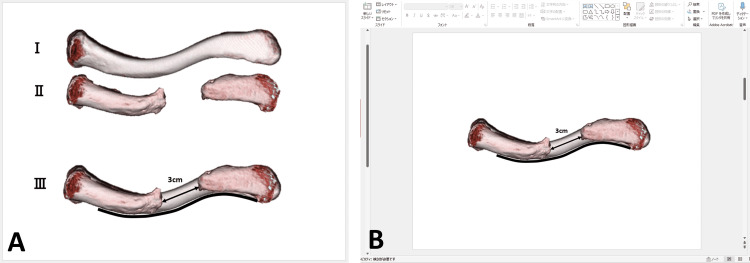
Creating a two-dimensional actual-size template The backgrounds of the images were removed. The image of the normal clavicle was transformed into a mirror image (A.I). Next, the images of the nonunion fragments (A.II) were overlaid on the mirror-imaged normal clavicle, positioning the nonunion fragment images in front of it (A.III) to measure the size of the bone defect (3 cm). The actual-size template was created by printing the slide (B) on A4 paper, ensuring that “Scale to fit paper” was unchecked when selecting “Full-page slide size.”

Reconstructing the length and alignment of the clavicle in cases of large bone defects presents significant challenges. A 2D template was created to guide the clavicle's length and alignment, as shown in the Appendices (Figures 11-12). The 2D template of the actual-sized clavicle image was useful for assessing the bone defect size preoperatively and for contouring the plate to an anatomical shape intraoperatively, after printing and sterilizing the template with ethylene oxide gas. The anatomical length and alignment of the clavicle were confirmed to match the 2D template when compared to the three-dimensional (3D) CT images (Figure 9C) taken three months after the second stage of the Masquelet technique.

## Conclusions

A case of recalcitrant clavicle nonunion, following reconstructive surgery with a bone graft, was treated using the Masquelet technique. Clavicle length and alignment were reconstructed using locking plate fixation, guided by a 2D template created from an actual-sized clavicle image. The Masquelet technique can be performed using standard orthopedic surgical techniques without requiring specialized microsurgical techniques. An actual-sized 2D template is easier and more cost-effective to create than a 3D template.

Bone healing was achieved, and the patient experienced complete recovery with no pain and full shoulder function, demonstrating the effectiveness of this method in managing recalcitrant clavicle nonunion. The Masquelet technique is a promising option for the management of clavicular nonunion, offering effective results in terms of healing and functional recovery. However, further research, including multi-center studies and longer-term follow-up, is needed to validate its long-term outcomes and assess its performance in larger, more diverse patient populations.

## Appendices

A two-dimensional (2D) template was created to guide the clavicle length and alignment, using the following steps:

First, a three-dimensional (3D) image of both clavicles was generated from computed tomography (CT) scans using a Synapse Vincent Volume Analyzer (Fujifilm, Tokyo, Japan). Second, the 3D image of the normal clavicle was rotated along its longitudinal axis to obtain the widest view of the distal clavicle and then copied and pasted onto a slide using presentation software (Microsoft PowerPoint 2019; Microsoft® Corp., Redmond, WA, USA). Third, a 3D image of the nonunion clavicle fragments, under the same conditions, was copied and pasted onto the same slide. Fourth, the sizes of all images were adjusted to their actual dimensions based on the scale displayed using the tools in the presentation software. Finally, the image of the normal clavicle was mirrored, and the background was removed. The nonunion fragment images were positioned in front of the mirror image of the normal clavicle (Figures 11-12). The actual-size template was created by printing the slide (Figure 12B) on A4 paper, ensuring that “Scale to fit paper” was unchecked when selecting “Full-page slide size.”

## References

[1] Rowe CR (1968). An atlas of anatomy and treatment of midclavicular fractures. Clin Orthop Relat Res.

[2] Canadian Orthopaedic Trauma Society (2007). Nonoperative treatment compared with plate fixation of displaced midshaft clavicular fractures. A multicenter, randomized clinical trial. J Bone Joint Surg Am.

[3] Robinson CM, Goudie EB, Murray IR (2013). Open reduction and plate fixation versus nonoperative treatment for displaced midshaft clavicular fractures: a multicenter, randomized, controlled trial. J Bone Joint Surg Am.

[4] Ahrens PM, Garlick NI, Barber J, Tims EM (2017). The clavicle trial: a multicenter randomized controlled trial comparing operative with nonoperative treatment of displaced midshaft clavicle fractures. J Bone Joint Surg Am.

[5] Qin M, Zhao S, Guo W (2019). Open reduction and plate fixation compared with non-surgical treatment for displaced midshaft clavicle fracture: a meta-analysis of randomized clinical trials. Medicine (Baltimore).

[6] Fridberg M, Ban I, Issa Z, Krasheninnikoff M, Troelsen A (2013). Locking plate osteosynthesis of clavicle fractures: complication and reoperation rates in one hundred and five consecutive cases. Int Orthop.

[7] Andrade-Silva FB, Kojima KE, Joeris A, Santos Silva J, Mattar R Jr (2015). Single, superiorly placed reconstruction plate compared with flexible intramedullary nailing for midshaft clavicular fractures: a prospective, randomized controlled trial. J Bone Joint Surg Am.

[8] Woltz S, Duijff JW, Hoogendoorn JM, Rhemrev SJ, Breederveld RS, Schipper IB, Beeres FJ (2016). Reconstruction plates for midshaft clavicular fractures: a retrospective cohort study. Orthop Traumatol Surg Res.

[9] Meeuwis MA, Pull Ter Gunne AF, Verhofstad MH, van der Heijden FH (2017). Construct failure after open reduction and plate fixation of displaced midshaft clavicular fractures. Injury.

[10] Chiu YC, Huang KC, Shih CM, Lee KT, Chen KH, Hsu CE (2019). Comparison of implant failure rates of different plates for midshaft clavicular fractures based on fracture classifications. J Orthop Surg Res.

[11] Endrizzi DP, White RR, Babikian GM, Old AB (2008). Nonunion of the clavicle treated with plate fixation: a review of forty-seven consecutive cases. J Shoulder Elbow Surg.

[12] von Rüden C, Morgenstern M, Friederichs J (2016). Comparative study suggests that human bone morphogenetic proteins have no influence on the outcome of operative treatment of aseptic clavicle non-unions. Int Orthop.

[13] Wiss DA, Garlich JM (2021). Clavicle nonunion: plate and graft type do not affect healing rates-a single surgeon experience with 71 cases. J Shoulder Elbow Surg.

[14] Lenoir H, Williams T, Kerfant N, Robert M, Le Nen D (2013). Free vascularized fibular graft as a salvage procedure for large clavicular defect: a two cases report. Orthop Traumatol Surg Res.

[15] Jaloux C, Bettex Q, Levadoux M (2020). Free vascularized medial femoral condyle corticoperiosteal flap with non-vascularized iliac crest graft for the treatment of recalcitrant clavicle non-union. J Plast Reconstr Aesthet Surg.

[16] Masquelet AC, Begue T (2010). The concept of induced membrane for reconstruction of long bone defects. Orthop Clin North Am.

[17] Masquelet A, Kanakaris NK, Obert L, Stafford P, Giannoudis PV (2019). Bone repair using the Masquelet technique. J Bone Joint Surg Am.

[18] Alford AI, Nicolaou D, Hake M, McBride-Gagyi S (2021). Masquelet's induced membrane technique: review of current concepts and future directions. J Orthop Res.

[19] Zura R, Xiong Z, Einhorn T (2016). Epidemiology of fracture nonunion in 18 human bones. JAMA Surg.

[20] Barret H, Mas V, Boissinot T, Baltassat A, Mansat P, Bonnevialle N (2024). Satisfactory results in five patients with septic clavicle nonunion using the modified Masquelet technique and structural iliac crest autograft. J Shoulder Elbow Surg Int.

